# Familial Aggregation and Heritability of Myopia: A Local Population Survey in Shanxi, China

**DOI:** 10.1155/2021/4847112

**Published:** 2021-08-10

**Authors:** Qin Wang, Hao-Yu Bi, Chun-Fang Wang

**Affiliations:** ^1^Department of Ophthalmology, First Hospital of Shanxi Medical University, Taiyuan 030000, Shanxi, China; ^2^Department of Ophthalmology, Changzhi People′s Hospital, Changzhi 046000, Shanxi, China; ^3^Gaoping Fuming Eye Hospital, Gaoping 048400, Shanxi, China

## Abstract

**Purpose:**

To further determine the roles of environmental and genetic factors in the development of myopia, a comprehensive survey was performed. The guidance for myopia-susceptible people is established which might help prevent or delay the onset and development of myopia.

**Methods:**

1,852 students were recruited using the multistage sampling approach from the Gaoping county in Shanxi. The refractive status of students was examined using an autorefractometer, and the refractive status of students' first-degree relatives was collected using a well-designed questionnaire. Family aggregation of myopia was analyzed according to the myopic status of the students (nonmyopic or myopic group). The prevalence and heritability of myopia in students and their first-degree relatives were further explored by subdividing into mild, moderate, and high myopia groups. Significance analysis among each group was performed by the *χ*^2^ test using SPSS 25.0 software. Falconer's method was used to calculate the inheritability of myopia.

**Results:**

A total of 1,852 subjects were recruited in this study, and 1,813 subjects were finally included. The family aggregation of myopia in the myopic student group (34.7%) was significantly higher than that in the nonmyopic group (8.5%). The prevalence of mild, moderate, and high myopia in children (students and siblings) was higher than that in their parents. The rate of high myopia (6.33%) was significantly higher among students with one or both myopic parents than those without myopic parents (3.85%). The heritability of mild, moderate, and high myopia among parents-offspring was 3.72%, 20.47%, and 48.00%, respectively. The heritability of mild, moderate, and high myopia among siblings was 17.50%, 86.09%, and 78.75%, which is significantly higher than that among parents-offspring. In addition to genetic factors, extensive near-work time, higher education pressure, and minimal outdoor activities contribute significantly to mild and moderate myopia.

**Conclusions:**

Myopia is of high risk due to familial aggregation. Students with a family history of myopia are more likely to have high myopia than those without family history. The occurrence and development of high myopia are affected by both the genetic and environmental factors, which could either weaken or strengthen myopia. Therefore, students with a family history of myopia should pay close attention to their eye health to avoid the occurrence of myopia and the deepening of diopter, which may lead to high myopia and its related complications.

## 1. Introduction

Myopia is the most common refractive error of the eye in people. It is estimated that the population with myopia and high myopia will increase to 4.758 billion and 938 million globally by 2050 due to changes in lifestyle and behavior [1]. Mounting evidence suggests that the aetiology of myopia is associated with multiple factors, including genetic and environmental factors. Particularly, the feature of familial aggregation for myopia indicates the critical roles of genetics, possibly a polygenic gene, in disease development. Indeed, with the development of genetic engineering and molecular biology in recent years, several genome-wide association studies (GWASs) of myopia have been successfully conducted and numerous candidate loci and genes have been identified for the development of myopia [2]. At present, 12 high myopia gene loci (MYP1-3, MYP5, MYP11-13, and MYP15-19) and 6 myopia loci (MYP6-10 and MPY14) were confirmed [3–5]. Besides these genetic factors [6], social environment and living standards have also been reported in myopia occurrence [7]. In addition, retinal complications caused by high myopia are associated with visual impairment and blindness [8]. To date, studies on heritability of myopia are mostly focused on high myopia population, but not on the mild or moderate myopia. We speculate that mild or moderate myopia is also caused by complicated factors, possibly due to interaction between the genetic and environmental factors. In this study, we assessed the susceptibility of myopia by calculating heritability to count genetic and environmental factors. Furthermore, the proposed guide of daily behaviors and habits for the susceptible group is established to possibly forestall or even prevent the onset of myopia.

## 2. Methods

### 2.1. Study Population

**Figure 1:**
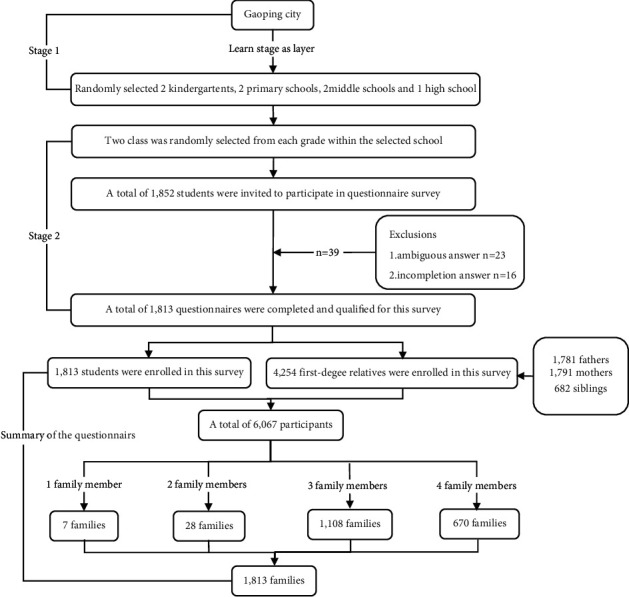
Flow diagram of sampling participants.

A multistage sampling method was applied in this study. In the first stage, subjects from 2 kindergartens, 2 primary schools, 2 middle schools, and one high school were randomly selected by stratified sampling with the learning stage as the layer. In the second stage, stratified cluster sampling was used to select two classes randomly from each grade of each school. Due to poor cooperation in various inspections among the lower-grade students (age < 5 years), only high-grade students were selected in kindergarten. A total of 1,852 students were invited to this study (Figure 1).

### 2.2. Questionnaire

A questionnaire was designed to investigate parents' and siblings' diopters and the diopter of spectacles according to the tenets of the Declaration of Helsinki. The purpose and method of the research was informed to parents and students before their consent was obtained. A pretest about the content of the questionnaire was evaluated for its feasibility, and some modifications and complements were done accordingly based on pretest results. The questionnaire data were collected by well-trained technicians with the help of parents and students to ensure the latest information of each family members.

### 2.3. Eye Examinations and Interpretation

Eye examinations, including uncorrected visual acuity, corrected visual acuity, and diopter, were performed for each student. The diopter of refractive status for each enrolled subject was examined with cycloplegia. In brief, tropicamide eye drops were used for cycloplegia. Four drops were instilled in the inferior conjunctival cul-de-sac at an interval of five minutes. If the pupillary light reflex was still present after 20 minutes, five drops were administered. Cycloplegia was considered as at completion if the pupil was dilated to 6 mm or more and there was no pupillary light reflex. The measurement of the diopter was performed using an autorefractometer (RM-8900; Topcon, Tokyo, Japan) for both eyes. Diopter is equal to spherical equivalent, which is the sum of the full spheres and 1/2 cylinders. According to spherical equivalent, myopia is defined as SE ≤ −0.50 D, which is further divided into three categories: mild myopia: refractive error less than −3.00 D; moderate myopia: refractive error greater than −3.00 D and less than −6.00 D; high myopia: refractive error equal or greater than −6.00 D. Familial aggregation is defined as follows: in a family, there are at least two family members with positive corresponding indicators.

### 2.4. Statistical Analysis

Heritability was measured using Falconer's regression. The *X* and *a*_*c*_ of normal distribution could be examined according to the prevalence of myopia among first-degree relatives of patients with myopia. Heritability (*h*^2^) was expressed as *b*/*r*, where *b* is the correlation coefficient and expressed as (*X*_*c*_ − *X*_*r*_)/*a*_*c*_ (*X*_*c*_ is the mean of the prevalence of immediate family among the nonmyopic student group, *X*_*r*_ is the mean of the prevalence of immediate family among the myopic student group, and *a*_*c*_ is the variance of the prevalence of immediate family among nonmyopic student group). *r* is the kinship correlation and equal to 0.5 in this case. In this study, we use percent (%) to estimate the role of pathogenic genes in polygenic genetic diseases. 70%–80% heritability indicates that the genetic factor is a dominant reason for the disease, while 30%–40% heritability indicates that the environmental factor is more important than genetic factor for onset of the disease.

SPSS25.0 software was used for statistical analysis. The adoption rate or composition ratio of classified data such as prevalence rate of myopia and familial aggregation of students and first-degree relatives was analyzed. The Chi-square test was used to test whether or not the expected relationship exists. A *P* value less than 0.05 was considered as significance between/among groups.

## 3. Results

### 3.1. Summary of the Questionnaires

A total of 1,852 students were invited to participate in the questionnaire survey. 39 questionnaires were invalid due to either ambiguous answers (23 questionnaires) or incompletion (16 questionnaires). A total of 1,813 questionnaires were completed and qualified for our study. A total of 4,254 first-degree relatives were included in the analysis, with 1,781 fathers, 1,791 mothers, and 682 siblings. In the end, 6,067 participants from 1,813 families were included in this study. Seven families had 1 immediate family member. 28 families had 2 immediate family members. 1,108 families had 3 immediate family members, and 670 families had 4 immediate family members in this study (Figure 1).

### 3.2. Results of Eye Measurements

**Figure 2:**
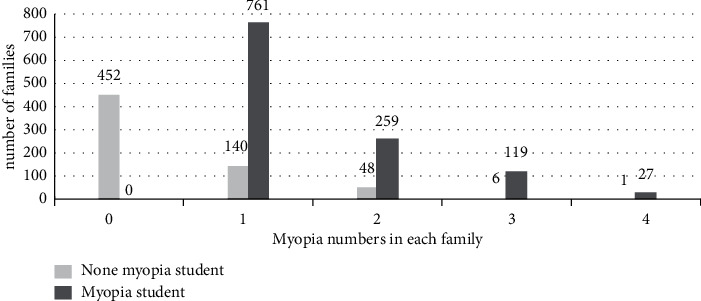
Distribution of family aggregation of myopia in this study.

A total of 1,813 families participated in this study. According to the status of myopia, the students are divided into nonmyopic group and myopic group. In the nonmyopic student group, 452 families had nonmyopic patients, 140 families had only 1 myopic patient, 48 families had 2 myopic patients, 6 families had 3 myopic patients, and 1 family had 4 myopic patients. However, in the myopic student group, 761 families had only 1 myopic patient, 259 families had 2 myopic patients, 119 families had 3 myopic patients, and 27 families had 4 myopic patients (Figure 2).

A total of 460 families have familial aggregation of myopia, among which 55 families among the nonmyopic student group had familial aggregation, accounting for 8.50% of the nonmyopic student group. 405 families in the myopic student group had familial aggregation, accounting for 34.73% of the students with myopia, which was significantly higher than that of the students without myopia (*χ*2 = 151.235, *P* < 0.001) (Table 1).

### 3.3. Effect of First-Degree Relatives' Refractive Status on Each Subgroup

According to the refractive status, the participants were divided into four subgroups: no myopia, mild myopia, moderate myopia, and high myopia. The number of students and their first-degree relatives and the proportion in each subgroup are shown in Table 2. We found that students and their siblings had higher myopic prevalence than their parents in each subgroup. In addition, myopia status of parents and students is shown in Table 3. For students who had mild myopia (*χ*2 = 1.932, *P*=0.164) or moderate myopia (*χ*2 = 1.709, *P*=0.191), the percentage between myopic parents and nonmyopic parents was nearly equal. While in the high myopia group, the percentage of myopic parents was higher than of nonmyopic parents (*χ*2 = 5.058, *P*=0.025). There was statistical evidence of parents' refractive status effect on students.

### 3.4. Myopia Heritability of Parents-Offspring and Siblings

Tables 4 and 5 show the myopia heritability of parents-offspring and siblings. High myopia shows the highest heritability for parents-offspring heritability (48.00%). Compared with parents-offspring heritability, sibling heritability was obvious. Interestingly, compared with the parents-offspring myopia heritability, the myopia heritability among siblings was higher than that between parents and children in the mild, moderate, and high myopia group.

## 4. Discussion

Myopia is the most serious type of refractive error that causes visual impairment [9]. The severity and early occurrence of myopia not only damage the physical and mental health of children and adolescents and affect their future education and career choice but also bring great challenges and economic burdens to the society, especially the medical and health industry. Previous genetic epidemiological research on myopia shows that myopia was influenced by genetic factors and familial aggregation. According to analysis of myopia in family data, we found family aggregation among myopic students is more obvious than in nonmyopic students, indicating the polygenic effect of the disease. Farbrother et al. [10] also proposed that myopia has a family aggregation trend in their research, demonstrating the importance of genetic factor in the onset of myopia.

After investigating the students and their relatives, we found prevalence of myopia is higher among students than parental myopia at any myopia stage. One possible reason for this is the difference in the growth environment. As for parents, they lived in an agrarian lifestyle when they were young and did not have many chances to engage in modern technologies such as computer, television, and video games. Pan et al. [11] reported the prevalence of myopia was higher in second-generation immigrants than first-generation immigrants according to their study on 3400 Singaporean Indians aged over 40 years. These studies show that genetic factors will not significantly change within several generations and the higher prevalence of myopia in the second generation is not largely influenced by genetic factors but environmental factors.

Several studies [12, 13] reported myopic parents have great influence on their offspring. In a family of both parents having myopia, their children showed a higher risk to harbor myopia than in family that only has one myopic parent. This is in consistent with our results in high myopia family. While in the mild or moderate myopia family, we found there was no significant association with high risk. The possible reason for this discrepancy might relate with the effects of environmental factors on mild or moderate myopia.

With the prevalence of myopia across the world, an increasing number of research studies focus on the reason of myopia [14]. The interaction of the genetic and environmental factors is important in the development of myopia. Although the exact mechanism of how environmental factors influence myopia in action is still unsettled, the importance of these factors in myopia has been continuously verified, including extensive near-work time, higher education pressure, and minimal outdoor activities [15–17]. Interestingly, under nearly the same environment, there is a higher risk to have myopia for some individuals or family than others [18]. This phenomenon is closely related with genetic susceptibility of myopia. Genes determine an individual's susceptibility to environmental factors [19]. With the increasing prevalence of myopia, evaluating the susceptibility of myopia is becoming important. Myopia heritability is a good index for susceptibility. In our study, the heritability in mild, moderate, and high myopia is 3.72%, 20.47%, and 48.00%, respectively, indicating that environmental factors have greater influence on mild and moderate myopia, while the genetic factor is more obvious for high myopia. And, myopia is caused by an interplay between genetic factor and environmental factor. With potential myopia genes in family, offspring has higher possibility to have myopia if they do not have good lifestyle and habits. Hwang et al. [20] discovered that the heritability of mild myopia and high myopia are 44.3% and 68.9% in Koreans, which is higher than our result. The possible reasons include different lifestyles between two races, learning environment and social environment, or different age groups of the research object. Dirani et al. [21] recruited 345 pairs of identical twins and 267 pairs of nonidentical twins and found that myopia genetic rate was 88% in men and 75% in women. Heritabilities of brother-brother and sister-sister siblings in the Old Order Amish are 60% and 64%, respectively [22]. Compared with our results, the heritability varies among different regions due to different population samples and estimation methods, but all the results show that genetic factors contribute to the occurrence of myopia.

In addition, sib-sib heritability of myopia is obviously higher than parents-offspring heritability in our study. It is not a surprise because siblings of close age normally have the same family environment and similar behaviors. Clustering effect cannot be ignored since more and more young generations have myopia. Morgan and Rose [23] conducted a research on the varying myopia heritability in sib-sib and parents-offspring with social environment change. They suggested that sib-sib heritability remains high level no matter the changes of social environment, which is consistent with an early study by Guggenheim et al. [24].

Siblings and parents-offspring share similar genetic background. If only considering the effects of genetic factors on myopia, the heritability should be almost equal between siblings and parents-offspring. However, both our study and past research found that sib-sib heritability is higher than parents-offspring heritability, demonstrating that there is interplay between environmental factors and genetic factors. Although the genetic background is similar between siblings and parents-offspring, the siblings in the same era have more identical social environment, family environment, and behavior habits, which ultimately lead to the heritability of myopia between siblings being significantly higher than that between parents and children. All of these studies indicate that environmental factors cannot be ignored in myopia formation process.

Our study with the multistage survey method involving participants in all age groups avoids the influence of excessive age gap. In addition, this study takes the family as a unit and is an ideal choice for studying heritability and family aggregation. At the same time, the economic and cultural environment of the parents is more inclined to the agricultural era, which is in sharp contrast with the social environment of contemporary children. It is helpful to study the role of environmental factors in the development of myopia. However, there are some limitations in this study. First, the diopter of first-degree relatives was obtained by a questionnaire, which may induce bias. Second, due to the limitation of external conditions, the diopter of children was obtained with tropicamide but not atropine, which might result in a small measurement deviation of the diopter results. Therefore, the relevant experimental procedures need to be improved in further studies.

In conclusion, our study provides additional evidence for myopia as a polygenic disease influenced by both gene and environment. Heritability between family member and common environment can either weaken or strengthen myopia. Therefore, actions on better lifestyle for children are important in order to avoid the onset and development of myopia.

## Figures and Tables

**Table 1:** Family aggregation of myopia in this study.

Characteristics	Without family aggregation (*N*, %)	With family aggregation (*N*, %)	*χ*2	*P*
Nonmyopic student	592 (91.50%)	55 (8.50)	151.235	<0.001
Myopic student	761 (65.26)	405 (34.73)		

*N* = number of families; % = *N*/the total *N*.

**Table 2:** The effect of first-degree relatives' refractive status on each subgroup.

	No myopia (*N*, %)	Mild myopia (*N*, %)	Moderate myopia (*N*, %)	High myopia (*N*, %)	In total (*N*, %)
Fathers	1503 (84.39%)	150 (8.42%)	104 (5.84%)	24 (1.34%)	1781 (100%)
Mothers	1428 (79.73%)	199 (11.11%)	123 (6.87%)	41 (2.29%)	1791 (100%)
Siblings	494 (72.43%)	110 (16.14%)	61 (8.94%)	17 (2.49%)	682 (100%)
Students	647 (35.69%)	721 (39.77%)	363 (20.02%)	82 (4.52%)	1813 (100%)

*N* = number of participants; % = *N* in each subgroup/the total *N* in each category.

**Table 3:** Comparison of myopia status between parents and students.

Status	No myopia	Mild myopia	Moderate myopia	High myopia
Myopic parents	169 (34.49%)	182 (37.14%)	108 (22.04%)	31 (6.33%)
Nonmyopic parents	478 (36.18%)	539 (40.74%)	25 5(19.27)	51 (3.85%)
*χ*2	0.419	1.932	1.709	5.058
*P*	0.517	0.164	0.191	0.025

**Table 4:** Myopia heritability of students and parents.

Student status	Parents	Myopic parents	*q* (%)	*x*	*a*	*b*	*h*^*2*^ (%)
No myopia	1267	210	16.57	0.974	1.504		
Mild myopia	1425	245	17.19	0.946	1.482	0.019	3.72
Moderate myopia	688	142	20.64	0.820	1.383	0.102	20.48
High myopia	162	44	27.16	0.613	1.225	0.240	48.00

**Table 5:** Myopia heritability among siblings.

Student status	Siblings	Myopic siblings	*q* (%)	*x*	*a*	*b*	*h*^*2*^ (%)
No myopia	181	35	19.33	0.863	1.417		
Mild myopia	282	66	23.40	0.739	1.320	0.088	17.50
Moderate myopia	172	69	40.12	0.253	0.966	0.433	86.09
High myopia	48	18	37.50	0.305	1.002	0.394	78.75

## Data Availability

All data generated and used during the study are included within the article.
